# Variations in subjective definitions of everyday situations as intergroup contact

**DOI:** 10.1111/bjso.12372

**Published:** 2020-02-24

**Authors:** Tina F. Keil, Miriam Koschate

**Affiliations:** ^1^ University of Exeter UK

**Keywords:** contact measurement, intergroup contact, negative contact, online contact, subjective definition

## Abstract

Intergroup contact encompasses a wide range of contact situations. Yet, how ‘contact’ is conceptualized by those involved has rarely been examined. We argue that understanding the range of subjective definitions of contact is important for intergroup contact measurement and wider impact work. In Study 1, 17 participants completed a 3‐day diary and a semi‐structured interview about their experiences of contact with other nationalities. We examined the threshold at which encounters are subjectively defined as intergroup contact. Results showed that subjective definitions of intergroup contact were disparate and diverse, particularly when contact was fleeting or online. In Study 2, we asked a British sample (*N* = 498) to rate the extent to which 67 different contact scenarios with non‐British people represented ‘intergroup contact’. Findings show that contact situations which diverge from positive, verbal, face‐to‐face encounters, such as negative contact or online contact, were less likely to be understood as contact, with strong variation in ratings. The extent to which situations were seen as contact was positively correlated with the amount of self‐reported intergroup contact. Together, these findings demonstrate the need to recognize and account for the variability in subjective definitions of contact, which ultimately shape self‐reports of intergroup contact.

## Background

Everyday interactions with people of diverse ethnic, cultural, and social backgrounds are an increasingly common part of our daily lives (Coleman, 2013; Devine, Evett, & Vasquez‐Suson, 1996; Jivary, 2012; Vertovec, 2010). Many of these encounters are short and banal in nature and have received relatively little attention from past research on intergroup contact. Following Allport's (1954/1979) contact hypothesis and Pettigrew’s (1997) paper on the importance of friendship contact, much of the intergroup contact literature has focused on optimal forms of contact (for meta‐analyses, see Davies, Tropp, Aron, Pettigrew, & Wright, 2011; Lemmer & Wagner, 2015; Pettigrew & Tropp, 2006). However, more recent research has started to look at less clearly defined forms of intergroup contact, such as online contact (e.g., Amichai‐Hamburger, Hasler, & Shani‐Sherman, 2015), negative contact (e.g., Barlow *et al.*, 2012; Graf, Paolini, & Rubin, 2014), and naturally occurring contact in public spaces (e.g., Dixon *et al.*, 2020; Thomsen & Rafiqi, 2018). The question now arises whether these forms of contact are universally defined as constituting contact.

A shared understanding of what we mean by contact is essential for both accurate measurement and effective communication with stakeholders. The vast majority of intergroup contact studies use broad self‐report measures that aim to capture a wide variety of contact experiences (Pettigrew, 2008). However, such measures rely heavily on a shared definition of contact between researchers and participants, that is, a shared understanding of which situations should be considered as contact. Similarly, when communicating with stakeholders such as community leaders, charities, or institutions (e.g., schools) about interventions to reduce prejudice, a shared understanding is essential for a successful implementation. The aim of this study is to help researchers understand how lay people subjectively define which situations constitute ‘intergroup contact’ in everyday encounters, how varied such definitions and conceptualizations are, and how they relate to self‐reported intergroup contact quantity.

By exploring a lay understanding of intergroup contact, this paper therefore contributes to the literature in two important ways: (1) it helps researchers and practitioners to design studies and interventions that reflect a shared understanding of what contact means, rather than having to rely on assumptions; (2) it provides an initial examination of the validity of standard contact measures by testing how strongly these are affected by differing subjective definitions of contact.

### Defining intergroup contact

Early intergroup contact researchers, such as Cook and Selltiz (1955), pointed out the importance of asking ‘What is contact?’ Since then, attempts by researchers to define intergroup contact show that it is difficult to capture the multitude of encounters with members of other groups. Sherif and Sherif (1953), for instance, noted that contact is ‘a “blank term” for many different types of interaction’ (p. 221). For their meta‐analysis of intergroup contact effects, Pettigrew and Tropp (2006) defined intergroup contact as an ‘actual face‐to‐face interaction between members of clearly defined groups’ (p. 754). In the context of friendship and acquaintanceship contact, such a definition appears relatively unproblematic, in the sense that most individuals are likely to define a face‐to‐face encounter with an outgroup friend or acquaintance as ‘contact’.

In recent years, however, contact research has gone well‐beyond face‐to‐face contact with well‐known others: Intergroup contact now encompasses indirect, vicarious, and imagined contact where the individual is not experiencing any face‐to‐face interaction themselves (see Brown & Paterson, 2016 for a critical review). Even when a person experiences contact directly, nowadays this might be by means other than a face‐to‐face interaction, given the continuous rise of online social interactions (Wellman, Quan Haase, Witte, & Hampton, 2001). Also, research on effects of negative intergroup contact (Barlow *et al.*, 2012) has increased interest in everyday, superficial contact experiences that go beyond positive, face‐to‐face interactions with well‐known others (e.g., Thomsen & Rafiqi, 2018). When investigating superficial contact, a clear definition of what intergroup contact entails becomes vital. For instance, Pettigrew and Tropp (2006) argued that proximity or mere opportunity do not constitute intergroup contact, instead focusing on interactions (see also Cook & Selltiz, 1955 for a similar argument). But where does a social *interaction* begin?

Goffman (1963) suggested that a social interaction can be either unfocused or focused. He considered mutual acknowledgement, such as a nod, smile, or greeting, to be an unfocused interaction. In contrast, a conversation that goes beyond simple role‐based interactions (e.g., in service settings) is considered a focused interaction. Similarly, Nezlek (1993) uses mutual acknowledgement as the defining criterion by saying that a social interaction is ‘any encounter with at least one other person in which the participants attended to one another and adjusted their behavior in response to one another’ (p. 932; see also Wheeler & Nezlek, 1977). By this definition, even relatively fleeting exchanges, such as eye contact, if mutually acknowledged, would constitute a social interaction and therefore contact.

However, as Dixon, Durrheim and Tredoux (2005) point out, lay constructions of contact are likely to be diverse and disparate. Academic definitions may be quite far removed from the experiences and subjective definitions of lay people. To date, only a few studies have examined how ordinary people conceptualize contact that they experienced in an intergroup context.

One such study is an analysis by Dixon and Reicher (1997) of the contact experiences of White property owners with a Black homeless community in South Africa. Using semi‐structured interviews and discourse analysis, they focused on the narratives of White settlers and the context‐dependent nature in which social categories (e.g., Black/White, squatter/property–owner, and transient/permanent) were used in these accounts. Although they did not examine in detail which situations were conceptualized as contact, the study highlights that subjective experiences of contact are dynamic and contrasting in nature and that a researcher‐led definition of contact – particularly in quantitative terms – is removed from the multifaceted experiences of participants.

A more recent longitudinal study examined students’ definitions and accounts of everyday intercultural contact in a multicultural university setting (Halualani, 2008, 2010). Using a large number of in‐depth interviews, the study focused on how participants framed intercultural interactions. The initial thematic analysis showed that the majority of participants could not recall specific interactions, as these were too frequent on the multicultural campus of their university. Interactions that were reported also tended to exclude closer forms of contact, which were often categorized as being ‘just friends’, and therefore not part of participants’ concept of intergroup contact.

Similar to Dixon and Reicher (1997), Halualani (2008) emphasizes the differences in the construction of the ‘other’. Both studies suggest that the ‘intergroup’ aspect of the intergroup contact definition is constructed differently by lay people than by intergroup contact researchers who tend to study clearly defined (demographic) groups (e.g., by ethnicity, sexuality, age). The problem of whether intergroup contact is indeed experienced as ‘intergroup’ or as ‘interpersonal’, and the implications of different forms of social categorization for prejudice reduction, have been addressed by several researchers (e.g., Eller & Abrams, 2004; Hewstone & Brown, 1986; Pettigrew, 1998). In contrast, we are not aware of any study that has addressed the issue of when an encounter is considered as ‘contact’. Correspondingly, this research focuses on people’s own constructions and subjective definitions of contact. Our enquiry begins with two very basic questions: ‘Which situations are regarded as contact?’ and ‘How heterogeneous are subjective definitions of the boundaries of intergroup contact?’

### Measurement of intergroup contact

The question of when a situation is defined as intergroup contact has implications for the measurement of intergroup contact in self‐reports. Intergroup contact research is largely conducted in terms of self‐report surveys (81%, according to Hewstone, Judd, & Sharp, 2010; Pettigrew, 2008). Typically, items assess both the quantity and quality of contact. Contact quantity is usually assessed in broad terms by measuring the amount of outgroup individuals that participants are in contact with, the frequency of intergroup contact (usually within a specified time period), or the extent of intergroup contact (Lolliot *et al.*, 2015). Typical items are as follows: ‘How much contact do you have with [members of group x]?’, ‘How often do you have contact with [members of group x]?’ (e.g., Aberson & Haag, 2007; Brown, Maras, Masser, Vivian, & Hewstone, 2001; Miller, 2004; Tam *et al.*, 2007; Voci & Hewstone, 2003). Studies do not tend to provide a definition to participants of which situations to include and which situations they should not consider as a contact experience. While items are intentionally broadly formulated to capture different types of contact, they leave it up to the participant to interpret the inclusiveness of what they should report (Elson, 2017; Foddy, 2003; Kalton & Schuman, 1982). In contrast, measures that are formulated in overly specific terms may lead to an underestimation of what the item(s) intend(ed) to gauge, while at the same time restricting the participants’ own construal and meaning of what they are asked to report on (see Mallet, Akimoto, & Oishi, 2016). If participants agree on which situations they consider to be contact, self‐report measurements are a valid form of assessment (Christ & Wagner, 2012). However, if subjective definitions are heterogeneous, those who consider more situations as contact are more likely to report a higher quantity of intergroup contact than those with more restrictive definitions of contact despite potentially having the same amount of attitude‐changing encounters with outgroup members. For example, if one person defines contact in terms of non‐verbal mutual acknowledgement (e.g., eye contact, smiling at each other, moving seats on a bus), whereas a second person only considers personal conversations as intergroup contact, the amount of self‐reported contact is likely to differ substantially – despite the actual number of contact encounters being identical. Such conceptual inflation would undermine the validity of standard self‐report contact measures.

Although the vast majority of intergroup contact studies use self‐report measures (Pettigrew, 2008), observational measures have also been used in two related research traditions: (de‐)segregation research (e.g., Alexander & Tredoux, 2010; Dixon & Durrheim, 2003; McKeown, Cairns, Stringer, & Rae, 2012) and the intergroup interaction literature (see MacInnis & Page‐Gould, 2015, for a discussion). Observational measures are not affected by participants’ subjective definition in the same way as self‐report measures. They are, nevertheless, influenced by what the experimenter defines to be contact and what can be reliably observed.

The (de‐)segregation literature uses physical and temporal proximity in naturally occurring contexts, particularly seating patterns (e.g., in school/university classrooms, dining halls, or in public spaces, such as beaches), as a proxy for intergroup contact. The main finding in this literature is that groups tend to self‐segregate rather than integrate in unstructured, naturally occurring situations. However, it has been argued that physical proximity is not a contact measure in and of itself as it does not assess whether any mutual acknowledgement or interaction has taken place (Pettigrew & Tropp, 2006). Instead, it assesses opportunity for contact, which in combination with self‐report measures, provides valuable insights into how intergroup contact can be fostered in diverse but self‐segregating societies (Dixon *et al.*, 2020; Schrieff, Tredoux, Finchilescu, & Dixon, 2010; Wagner, Christ, Pettigrew, & Wolf, 2006).

The social interaction literature observes structured or guided interactions between strangers in laboratory settings. These studies tend to find that intergroup interactions produce more negative outcomes such as discomfort, anxiety, and heightened levels of (identity) threat than intragroup interactions (MacInnis & Page‐Gould, 2015; see also Toosi, Babbitt, Ambady, & Sommers, 2012, for a meta‐analysis of interracial interactions). This appears to contrast with findings in the intergroup contact literature showing that contact increases empathy and reduces anxiety and threat (see Pettigrew & Tropp, 2008, for a meta‐analysis). However, it has been suggested that these differences might be due to several factors such as the length/regularity of interactions (once versus longer term), the context (artificial versus natural), and familiarity (strangers versus known others).

Taken together, findings with observational measures provide a much less positive picture of interactions between strangers than self‐report measures of intergroup contact generally do. Although several explanations exist that may account for these discrepancies, observational measures do not provide reassurance that self‐report measures are a valid form of assessment of intergroup contact, specifically with regard to contact with strangers.

### The current studies

In order to examine lay definitions of intergroup contact, we began with a qualitative exploration of the nature of people’s own constructions, definitions, and experiences of contact in an intergroup context (Study 1). Our approach differs from Dixon and Reicher (1997) and Halualani (2008, 2010) by focusing less on the construction of what ‘intergroup’ means, and more on what is considered ‘contact’. Hence, in Study 1, we explored how and to what extent people conceptualize contact.

Using a quantitative approach, Study 2 examines how much individuals differ in their subjective definitions of different types of encounters as constituting ‘intergroup contact’, and the relationship of the inclusiveness of their subjective contact definition with the quantity of contact they report retrospectively, testing the validity of standard contact measures.

## STUDY 1

## Method

### Participants

We deliberately sought a culturally heterogeneous sample to counteract participant selection bias and increase the scope, variety, and generalizability of accounts and experiences that can be gained from the sample (Dattalo, 2010; Robinson, 2014). Participants were recruited via the University’s participant recruitment system, posters, and snowball sampling. To gain a maximally diverse sample, participants interested in the study (*N* = 26) were pre‐screened and asked to indicate their nationality, gender, and age. The selection process was based on the maximum variance, calculated from the Z‐Scores of an individual’s age and the Culture ComPass™ (Hofstede, 2001; Hofstede, Hofstede & Minkov, 2010) score for their country of origin. This resulted in a final sample of 17 participants (71% female), aged between 18 and 27 years (*M* = 21.41, *SD* = 2.60; see Table 1).

**Table 1:** Participant demographics and number of contacts in the 3‐day diary

#	Pseudonym	Sex	Age	No. of contacts noted in diary		Nationality (Country)	Study subject or occupation
1	Fred	M	18	18	±	KH	Accounting
2	Jonathon	M	20	4		GB	English Literature
3	Anzehlika	F	22	14	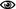	RU	Accounting
4	Julie*	F	27	16		KR	–
5	Alice	F	19	76	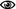	US	Drama
6	Charlie	M	23	20	±	CZ	Management
7	Courtney**	F	19	–		GB	English Literature
8	Mochi	M	23	14		GB	Arabic & Islamic
9	Lily	F	23	59		CN	Marketing
10	Emma	F	20	48	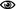 ±	CH	Psychology
11	Jane	F	23	16	±	BG	Management
12	Grace	F	18	8	±	GB	Psychology
13	Eve*	F	25	50	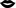 ±	GR	Research Assistant
14	Kate	F	19	22		BG	Marketing
15	Catherine	F	20	46	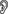 ±	JP	Geography
16	Red**	F	21	–		IN	Accounting
17	Ryan	M	24	22	±	SG	Sociology

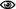
 reported eye contact in diary; 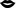 reported smiling as contact in diary; 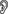 reported overhearing conversation as contact in diary; ± count is approximate, as diary entries partially unclear.

* Non‐student

** Did not provide diary after interview.

#### Design and procedure

The study employed a mixture of two classic ethnographic methods: an elicitation diary task (Carter & Mankoff, 2005) and a subsequent semi‐structured interview. The study was approved by the ethics committee at the Department of Psychology, University of Exeter. Informed consent was gained prior to participation.

#### Diary task

We asked participants to record all contacts with anyone of a different nationality to their own over the course of three consecutive days, with one of the three days being a weekend or bank holiday. We told participants to record such contacts in any way they preferred but that they should be as detailed as possible (see Appendix S1 for exact wording). Initially, we had not planned on collecting the diaries for analysis, as their primary purpose was to aid participants in the recall of their encounters during the interview. Nevertheless, we did ask participants after the interviews if they would be willing to provide us with their diaries. Diary formats were very varied (ranging from a simple tally list, screenshots from a mobile phone note app, basic handwritten notes, and longer story‐like descriptions, to detailed listing of each contact in an Excel sheet). The diaries were not included in the thematic analysis. We did, however, summate the number of contacts recorded in the diaries, as well as noting references to different types of contact (see Table 1).

#### Interviews

After completion of the diary task, participants took part in an open‐ended, semi‐structured interview. A basic topic guide was used to structure the interviews loosely. Although focusing first and foremost on the participants’ experiences of the intergroup contacts encountered during the 3‐day diary task, more general views and opinions about contact were not discouraged. On average, the interviews had a duration of about 40 min (*Min* = 32 min., *Max *= 70 min., *M *= 41.53, *SD *= 9.70). Upon completion of the interview, participants had the opportunity to choose a pseudonym, were given a remuneration of £15 each, and were debriefed.

### Analytic procedure

The audio‐recorded interviews were transcribed true verbatim,1Complete interview transcripts and coding files are available on OSF: https://doi.org/10.17605/osf.io/u5qj7. using simple transcription rules as outlined by Kuckartz, Dresing, Rädiker and Stefer (2008). Possibly identifying information, such as names of other persons, companies, or specific locations, was anonymized during transcription. Simple transcription symbols were inserted where appropriate during the transcription process.2Extract notations: Words in square brackets indicate insertion of an omitted word. Dots in square brackets indicate an excluded section of the original sentence. Vocalism, such as laughing, and non‐verbal expressions were added in parentheses. Slashes indicate a sudden break or abrupt end to a sentence.


The interview transcripts were analysed in a recursive, iterative, and interpretative cycle, using the six‐step process outlined by Braun and Clarke (2006, 2013). The steps involved were as follows: (1) familiarization with the data, (2) generation of initial codes, (3) searching for themes, (4) reviewing of themes, (5) defining themes, and (6) producing the report. This process enabled the organization and reduction of thick descriptive data.

The interviews were analysed at a semantic explicit level (Boyatzis, 1998; Braun & Clarke, 2006). We adopted an inductive approach during analysis, producing themes that arose from the underlying data, rather than from an *a priori* framework (Blanche, Durrheim, & Kelly, 2006).

MAXQDA, a qualitative data analysis software package, was used to assist the coding process. Words, paragraphs and/or sections of interest to the research focus were highlighted and assigned to initial codes. In a review process, differently named primary codes with similar or identical meanings were merged. Renamed codes were cross‐checked across all interviews to ensure that these still matched with the meaning of the initial code. This process resulted in a final sum of 937 codes (assigned to 1,981 different words, sentences, or paragraphs from all 17 interviews). Thematically and logically related codes were iteratively and systematically reviewed and grouped into suitable categories (484 with two sublevels)3There were 412 codes in sublevel one, and four codes in sublevel two. Also included were codes for activities, locations, and the nationality of the contact partners. and in turn, assigned to 38 higher‐order categories. The process was repeated until we were satisfied that the data had been sufficiently coded and categorized. Finally, prospective themes and subthemes within the boundaries of the given research focus were identified.

## Results

In total, participants originating from 13 different countries reported contact interactions with people from 54 different nationalities. Four main themes, situated within the context of our research focus, were derived from the data. While the fourth theme (Inhibiting and Promoting Factors) revealed differences in contact experiences, it did not relate to the question of which situations are considered contact and the theme is thus excluded here. The themes presented here are as follows: (1) Boundaries of Contact, (2) Quality of Contact, and (3) Locations of Contact.

### Theme 1: Boundaries of contact

This theme represents the idiosyncratic definitions of contact, and their boundaries, by lay participants. Answers are clustered in three subthemes: (1) mutual acknowledgement, (2) online contact, and (3) familiar contact. The following extracts illustrate a broad range of subjective definitions of contact. Further, they reveal the wide spectrum in which the categorical boundaries of different types of contact situations and relationships are situated. Through their diversity and disparity, the excerpts reveal an underlying sense of uncertainty and ambiguity, anchored both within the physicality of contact and its contemporary and manifold usage.

#### Mutual acknowledgement

One of the most common forms of mutual acknowledgement is eye contact, which plays an important role in human social interaction (Akechi *et al.*, 2013). When asked whether eye contact could be considered as contact, participants expressed a range of different views: ‘For me contact […] is when you make eye contact […] I went to {name of company} on one of those days [during the diary study], and I had an eye contact with the assistant, and I put it down here as one of the contacts’. (Anzehlika, 22, Russia). Similarly, Emma (20, Switzerland) describes several minimal contact encounters, including (without being prompted) eye contact: ‘We got to the tea place at three‐thirty, so we talked to the waiter. And then, obviously, I had contact with a lot of people on the way to town. I reckon about 12 actual eye contacts’. In contrast, Lily (23, China), while acknowledging eye contact as part of a contact event (e.g., during a conversation), clearly rejects it as a distinct form of contact:

I Good, okay. So, were there any other kind of contacts that you had, that you can think of during those three days, that you had any kind of situations? Even quite trivial, you know, kind of contacts? Maybe, for example, that you noticed somebody on the street, and you had eye contact. Would you actually consider that as a contact?

P Oh, eye contact? No, (chuckles). No, I thought maybe conversation is more like a contact […] If it is eye contact, it is not a contact […] I mean definitely we have eye contact during a conversation but if it was just someone staring at you, or [you] stare back, [then] I don’t think its contact.

Charlie (23, Czech Republic) and Courtney (19, Great Britain) both expressed uncertainty about eye contact as contact and suggested that eye contact needs to be complemented by something more than just a visual cue: ‘If you speak […] you make eye contact […], make a facial expression, and anything like that, it’s still contact, even though it’s really a small contact. Yeah, I don’t know’. (Courtney). According to Charlie, non‐verbal behaviour that evokes emotions and is reciprocal can be considered contact:

I You mentioned before, when you were listening to the father and son; did you make eye contact, did you look up at them? Or did you //

P I did look at them, but we hadn’t had any eye contact.

I They didn’t see you?

P Yeah. Maybe they saw me, but there wasn’t any eye contact, like direct eye contact.

I So, […] would you consider […] that a contact? Even though you don’t know them, and you wouldn’t talk to them?

P […] Sometimes, you just go out on the street, you see someone who is smiling from ear to ear, you smile to them as well. But yeah, I would consider that a contact if there’s some kind of interaction. If we just pass without anything, then I wouldn’t consider it as contact.

Some participants challenged whether mutual acknowledgements are enough to constitute contact. For instance, for Lily (23, China) contact needs to be meaningful: ‘[…] to say “hello” and “bye” and “thank you” to someone is also not contact […] if it doesn’t have any meaning in it, I wouldn’t regard it as a contact’. Similar to Lily’s subjective definition of contact, Catherine (20, Japan) also addresses the importance of meaning:

I And did you meet anybody new that you didn’t know before there?

P I think we kind of like passed the other housemates […] we were just like ‘Hi’, […] you know, going towards the bathroom and then they were coming out of their rooms […]

I Okay, but those // […] would say that’s contact for you?

P Well, just a passing ‘Hi’, I don’t know whether that’s contact […] it won’t be meaningful or […] substantial, significant kind of contact […]

I So, what would be [...] the minimal amount of meaningfulness that […]?

P I think at least sharing names […] just a few more questions, sentences and replies […] something [about] that person and then they know something about you.

In contrast, Jonathon (20, Great Britain) considers mutual acknowledgement as sufficiently constituting contact when describing a brief interaction with a ticket lady while visiting an exhibition:

I […] if [you] think back towards the last three days […] what would be one of the contacts you've had that is, I don't know, the least important, the least interesting, the most trivial?

P The most trivial […] that I would consider contact […] there was a Japanese woman working on the doors […]. That must have been less than a minute of conversation. Just like ‘Oh, hope you enjoyed the expo ‐ Thank you very much’, that sort of thing. I suppose that would have been the most trivial contact I would still call a contact.

#### Online contact

The following extracts illustrate an apparent dichotomy between contact that takes place in a shared physical space and online contact: When asked to recall his contact experiences on a particular day, Jonathon (20, Great Britain) asks: ‘Yesterday, does it count if it's online? Does it have to be physical?’ In contrast, when Emma (20, Switzerland) is asked why she hadn’t noted down any online contact, she points out that ‘[...] [I] often mistake contact as [contact with] humans and not just via the computer. But I know it is, now that I think about it’. Similarly, when Charlie (23, Czech Republic) is asked about online contact, he states: ‘To be honest, I haven’t really// I haven’t even thought that that would be contact?’ Later, he explains that although he is very active online, he strictly focused on the people he had met and had had conversations with, because he hadn’t ‘thought about the online environment as a place where you meet people, or [where] you do contacts’.

Much of the uncertainty around online contact hinges on the familiarity with the contact partner. For Charlie (23, Czech Republic), Facebook is reserved for people he has previously met face‐to‐face: ‘I don’t have anyone that I never met or who I never spoke to [in real life] on Facebook, so that’s really a personal platform for me’. Similarly, Julie (27, South Korea), when asked about online contact, points out: ‘For family or good friends it’s contact, but then, if there’s just a random person messaging you, it’s not always contact. I guess it depends a lot on your past relationship with that person’.

#### Familiar contact

Jane’s (23, Bulgaria) account of being in regular contact with her Czech boyfriend offers a stark contrast to the definition of brief contact encounters. To her, close and persistent contact oversteps the threshold of contact, rendering it to be invisible and beyond perception. When asked how she perceived the frequent contact with her boyfriend, Jane replied: ‘I haven’t thought of it as a contact really, yeah, it seems weird like […] because it’s not a one‐off, it’s constant – so it’s just, yeah, reality’.

### Theme 2: Quality of contact

This theme encapsulates examples of intergroup contact that show how contact in everyday environments can not only be meaningful and important but also have the capacity to affect bias, both positively and negatively. The following extracts focus on the quality of experience during everyday encounters, showing that relatively brief, superficial contact is recognized by some participants as an important experience.

#### Positive contact

Several participants reported positive intergroup contact experiences with strangers. For example, Lily (23, China) reported the following contact while travelling to Munich on the train:

P Yeah, I went to Germany at Christmas, and I spend a lot of time [there]; two months // two weeks there.

I Oh, wow.

P Yeah, before I went there, I felt that Germans [always] looked quite serious […] and then I met this 40‐50‐year‐old woman on the train […]. She sat there looking very serious – there were no spare seats. I wanted to sit down […]. She said something in German; I just took that to mean ‘Yes’ and sat down. She smiled and then we started talking […] she tried everything she could, to make me understand (laughs). Yeah, she was quite nice. I really liked her, and then she gave me a big hug.

Another example is Ryan’s (24, Singapore) experience of interacting with a Dutch bus driver, while on his way to a conference:

I So how did you get from the hotel to your conference (laughs)? […]

P […] he has this little soft toy in front of the bus, he was like ‘See, I’m very passionate about it’, and I was just laughing. He was really, really helpful and really friendly. It’s really nice because most of the bus trips that I’ve been on, the bus drivers have been really horrible.

#### Negative contact

The interviews also revealed some negative contact experiences that were considered to be contact. While at a conference, Ryan spoke of limited contact experiences with one of his roommates:

I But you didn’t […] have so much contact with them?

P I didn’t speak too much with them. Like the guy from the Middle East […] I wasn’t sure if he could actually take alcohol and if he was a Muslim […] and would arrive back at five in [the] morning, he was just going around drinking […] so, I didn’t really get along with him. I can’t even remember his name. It’s so bad (laughs).

Lily’s experience of interacting with a group of ‘foreigners’ during an assignment task at university is an example of how negative contact can also lead to contact avoidance:

I To […] get the most out of your time here, maybe?

P Yeah, […] I felt quite depressed last semester because we had a lot of assignments, and I don’t feel quite well [...] with those people. I got quite stressed […] in that group, you’ve got me and another Chinese girl and six foreigners, for me they are foreigners […] and then there’s this girl, sorry, but [she] is German (laughs) […] if I said I had an idea […] she just said, ‘Why can’t you tell me earlier? We’ve already decided.’ I just feel [that our] culture is too different, so we don’t talk to people like that, and we don’t do things like that.

#### Contact avoidance

Avoidance of contact was frequently achieved through non‐verbal strategies (e.g., consciously avoiding eye contact). Sometimes, such avoidance was reported as having directly resulted from previous negative experiences, social anxiety, or cultural differences in social norms. The following extracts illustrate the evasion of eye contact in order to avoid contact: Catherine (20, Japan) feels that strangers that make eye contact with her are ‘awkward’ and ‘intimidating’. It makes her ‘paranoid […], so I avoid it’. However, when asked if this could be due to cultural differences, she agrees but adds, ‘it must be a global thing’. Jane’s (23, Bulgaria) definition of eye contact as contact is similar, in that she feels that most people try to avoid eye contact, especially with strangers. However, she is unsure whether this is a conscious act, adding that she observes this, particularly while commuting, ‘especially in like buses and [on the] underground […], lots of them walk around with headphones on’.

### Theme 3: Locations of contact

The environment is an integral dimension of contact, contact opportunity, and its definition (Gustafson, 2001). Several participants referred to the location of contact as an important aspect of whether they consider a situation as contact. For example, Julie (27, South Korea) first reports that she feels ‘going shopping’ is not contact; she re‐evaluates her statement when she remembers that she regularly visits a small vegetable shop:

I Okay and what about shopping, did you go shopping?

P […] I think going to that vegetable shop is a contact […] I don’t know is that weird? Because […] it’s a little bit more personal going to little shops. They ask you, ‘How are you?’, whereas [in] {shop names} you say ‘Hi’, but you don’t really talk to them.

Similarly, Alice (19, US) notes that ‘a lot of times [in] smaller shops they’ll talk to you a bit more and ask you what you’re looking for and help you out or even strike up a conversation’. Another example is given by Jonathon, who describes how his experience of growing up in a rural area is related to his personal concept and definition of contact:

I And you mentioned you are from the {area of England}?

P I am from the middle of England, yeah, {name of town}

I Right, okay, so has […] the amount of contact increased4The question was asked in the context of Jonathan now living in a larger university town for his studies. a lot / that [you] have to people in general?

P Where I live was a very rural area […] most people were sort of an elderly age […]. There wasn’t much social contact to be found in the village […] I wouldn't have gone there by myself [...] if I wasn't meeting with someone. And, I suppose, this is where I get the idea that I need to meet with someone for it to be contact.

During the diary study, 63% of activities, spontaneously reported, took place in public settings, 19% in private settings, 11% at the workplace/place of study, and 7% online.

## Discussion

The present study, using a culturally diverse sample, explored subjective definitions and experiences of ordinary everyday intergroup contact. More specifically, the study sought to identify the minimal boundaries typically defined as being intergroup contact.

The TA revealed three major themes: The first theme, ‘Boundaries of Contact’, revealed subjective differences in the recognition of mutual acknowledgement as a minimum criterion for contact, uncertainty in the definition of online contact as contact, as well as difficulties to conceptualize highly familiar relationships as intergroup contact.

On the lower end of the spectrum of what is (still) defined as contact, some recognized *mutual acknowledgement* as being contact – others were less certain, and some rejected unfocused social interactions, such as eye contact or role‐based interactions (Goffman, 1963). As uncertainty decreased, subjective definitions of contact became more definitive, but also more restrictive, with an increasing number of conditions needing to be met before the situation could be considered contact (e.g., the inclusion of verbal components and ‘meaning’).

Noteworthy is also that *online contact* was not automatically equated with contact. This is surprising, considering that online communication has become an everyday medium, with recent findings suggesting that online intergroup contact is particularly well suited and practical in meeting the conditions set out by the contact hypothesis (see Amichai‐Hamburger & McKenna, 2006; Hasler & Amichai‐Hamburger, 2013). The findings suggest that online contact may not be a type of contact that is naturally salient and may not be automatically captured by the broad items typically used to assess contact in intergroup contact studies.

The subtheme *familiar contact* marks the boundary at the other end of the spectrum of what was defined as contact – being more akin to the experience of everyday reality than differentiable interactions. This finding also suggests that contact that is experienced as highly interpersonal is not considered in the lay definitions (and self‐reports) of intergroup contact.

The second theme, ‘*Quality of Contact*’, showed that ordinary, everyday interactions with outgroup members can be meaningful and important. Moreover, Theme 2 revealed both positive and negative contact experiences (Dixon, Levine, Reicher, & Durrheim, 2012; Laurence & Bentley, 2016) and included evasive tactics to minimize or avoid unwanted or anxiety evoking contact (see Kleinke, 1986; McDaniel, 1993; Stephan & Stephan, 1985).

Finally, the third theme, ‘*Locations of Contact*’, shows support for arguments that the location in which contact takes place is an integral dimension of social life and that the concept of (urban) space and its meaning is an object to which people orientate their actions (see Dixon, 2001; Jacobs, 2016; Mayblin *et al.*, 2015; Piekut & Valentine, 2017; Wessel, 2009). Participants provided a differentiated definition of contact in relation to its ‘locatedness’ (see Gustafson, 2001). Spaces of encounter are flavoured by their geographic and architectural characteristics (Jacobs, 2016). They can facilitate or inhibit contact and affect the subjective definition of what is seen as contact as well as shape its meaningfulness (Mayblin, Valentine, Kossak, & Schneider, 2015). Diaries also showed that the majority of encounters that were spontaneously considered to be contact occurred in public rather than private spaces or online. Locations provide diverse activity spaces often specialized towards particular everyday tasks (e.g., dancing at a club, drinking in a pub, eating in a restaurant, buying food in a supermarket, etc.; see also Bettencourt, Dixon, & Castro, 2019).

## STUDY 2

Following the qualitative enquiry, Study 2 aims to quantitatively examine the level of agreement of what is subjectively defined as contact. It also serves to replicate our findings from a culturally diverse sample with a culturally homogeneous majority group sample. More specifically, the intergroup contact literature largely focuses on majority groups, often White Europeans or White Americans. Assessing the extent to which a culturally and ethnically homogeneous sample differs in their subjective definition of contact is important as this will provide us with an initial test of whether conceptual inflation may be an issue in intergroup contact measurement in a relevant sample.

Based on Study 1 findings, we expect traditional forms of intergroup contact, such as positive, face‐to‐face interactions with outgroup friends and acquaintances, to be more clearly recognized as being intergroup contact than more fleeting interactions with strangers, or newer forms of contact, such as online interactions (Hypothesis 1). In addition, based on the problem of conceptual inflation, we predict that the extent to which various situations are subjectively defined to be contact is positively related to the amount of self‐reported intergroup contact (Hypothesis 2). Put differently, we expect participants with a wider subjective definition of contact to report a higher quantity of intergroup contact as they are likely to have more instances of contact to report that fits with their subjective definition.

### Method

#### Sample

Participants were recruited from various sources, such as the university’s participant recruitment system, several large Facebook special interest groups, and notices on dedicated participant recruitment websites. Thus, the participants were a mix of students and members of the general public. We aimed to recruit a sample of 500 participants to ensure sufficient power for an exploratory factor analysis of subjective definition of contact scenarios where we constructed scenarios in such a way as to increase the likelihood of an overdetermination of factors (i.e., several scenarios loading onto the same factor) and good communality (Hogarty, Hines, Kromrey, Ferron, & Mumford, 2005). A total of *N* = 525 British nationals over the age of 18 completed an online questionnaire. Twenty‐seven participants whose responses showed a statistically high level of acquiescence were excluded. This resulted in a final sample of *N *= 498 participants ((404 female, 81 %; 89 male, 18 %), two who reported another gender status, and three who did not declare their gender). The age of the sample ranged from 18 to 70 years (*M *= 32.99, *SD* = 13.87). The vast majority (91%) of participants consider themselves to be White British5This includes White British, English, Irish, Welsh, and Scottish with or eligible for UK citizenship. and the majority reported living in urban areas (63%). In terms of their highest level of education, our sample consisted of 2 (0.4%) participants without secondary education, 19 (4%) participants with secondary education (GCSE/O levels), 179 (36%) participants with post‐secondary education (A‐level and equivalent), 38 (8%) with vocational qualifications, 146 (29%) with an undergraduate degree, 94 (19%) with a postgraduate degree, and 17 (3%) with a PhD.

#### Procedure

After providing informed consent, participants were asked to complete an online questionnaire, which took approximately 15 min to complete. The questionnaire was administered through an open‐source online survey system.6Limesurvey™, installed on a dedicated secure web server (secure socket layer: SSL) located in the EU. The study was approved by the ethics committee of the Department of Psychology, University of Exeter. To avoid restricting the scope of possible contact situations, the outgroup was defined broadly as non‐British people. Items appeared in randomized order to avoid order effects and response sets. Participants were debriefed and given the choice of either being remunerated by gaining 0.5 course credits (for students) or by entering a prize draw with a chance of winning one of three £10 vouchers.

### Measures

The following measures are listed in order of their appearance in the online questionnaire and were adapted from previous studies of intergroup contact. Contact situations were mostly derived from the qualitative data in Study 1 (see Table S1).

#### Contact quantity

Contact quantity was assessed in line with previous survey‐based intergroup contact studies: six items, adapted from Islam and Hewstone (1993) and Viki, Culmer, Eller and Abrams (2006), were used to assess different contact settings and contexts (in general, neighbourhood, with friends, with family, with colleagues at work/ university, and in ordinary public settings, e.g., shopping). For example, ‘In the past, how much contact have you had with non‐British people in ordinary public settings (e.g., shopping)?’ Answers were assessed on a 7‐point scale (*0 = none at all, 6 = very much*) and displayed in random order. Reliability was good, with Cronbach’s α = .79.

To assess the number of outgroup contacts, we used two items with a numerical open answer format: Participants were asked to estimate the number of non‐British people with whom they had contact with during the last two weeks and the number of non‐British people with whom they were currently friends with (Halualani, Chitgopekar, Morrison, & Dodge, 2004; Koschate, Hofmann, & Schmitt, 2012). The two items were significantly but only moderately correlated (*r_s_* = .44, *p* < .001). We will therefore report results for each item separately and use Spearman’s ρ to reduce the influence of extreme values rather than excluding responses.

##### Subjective definition of contact

Participants were presented with 67 brief scenarios describing different relationships and situational contexts to capture the range of everyday scenarios that are subjectively defined as intergroup contact (see Table S1). These included both positive and negative scenarios, as well as non‐verbal and online interactions. An example item is: ‘How much do you consider being served by a non‐British person in a shop or supermarket as being contact?’ A 7‐point scale (*0 = not at all, 6 = very much*) was used for all items.

## Results

First, an exploratory factor analysis (EFA) was carried out on all the subjective definition of contact items. Subsequently, correlations between quantity of contact and subjective definition of contact factors were examined.

### Exploratory factor analysis

Using principal axis factoring, all 67 subjective definition of contact items showed their overall suitability for an EFA (with a good KMO of .96) and a highly significant Bartlett’s test of sphericity (χ^2^ (2,211, *N* = 498) = 23,605.33, *p* < .001). Initially, ten factors with Eigenvalues greater than one were extracted. A series of further EFAs were conducted to find an optimal and equal number of items that loaded most strongly (loadings > .50) and most cleanly (cross‐loadings < .32) onto separate factors. This process resulted in a final selection of 23 items with minimal cross‐factor loadings.

The EFA rendered several clearly differentiable contact types. A final 4‐factor solution (see Table 2) explained 62 % of the total variance (KMO = .90; Bartlett's test of sphericity, χ^2^ (253, *N* = 461) = 5460.91, *p* < .001; SVR = 20:1), with Eigenvalues, ranging from 7.24 to 1.39. Varimax rotation was chosen as it assumes all factors are uncorrelated. Other extraction methods and rotations were also tested (e.g., maximum likelihood and direct oblimin), which resulted in the same four factors. All items load above .50 with only three items cross‐loading marginally above .30. The four factors were labelled as (1) traditional contact, (2) superficial contact, (3) online contact, and (4) negative contact. Internal consistency of all four factors was good (see Table 2).

**Table 2:** Four‐factor solution for subjective definition of contact items (highest factor loadings in bold)

Items	F1	F2	F3	F4
Chatting on the phone to a non‐British family member	**.752**	.072	.046	−.001
Daily interaction with a non‐British friend or acquaintance	**.707**	.086	−.002	.036
Having an informal conversation with a non‐British person you know	**.695**	.253	.093	−.070
Having lunch at a cafeteria with a non‐British person	**.694**	.196	.054	.047
Going out to dinner with a non‐British person	**.676**	.054	−.024	.100
Chatting on the phone to a non‐British friend or acquaintance	**.654**	.167	.142	−.030
Visiting your non‐British friend’s or acquaintance's home	**.649**	−.030	−.075	.132
Skyping with a non‐British person	**.637**	.073	.125	.096
Being served by a non‐British person in a shop or supermarket	.167	**.840**	.230	.200
Purchasing a ticket from a non‐British person on a train or bus	.154	**.794**	.217	.225
Ordering something to drink or eat from a non‐British person	.176	**.695**	.290	.236
Receiving a marketing phone call from a non‐British person	.154	**.612**	.267	.200
Giving money to a non‐British person who is begging	.195	**.511**	.274	.280
‘Liking’ a post on Facebook/Twitter from a non‐British person	.058	.245	**.833**	.087
Sharing/retweeting a non‐British person's post on Facebook/Twitter	.021	.121	**.813**	.111
Commenting on a Facebook/Twitter post from a non‐British person	.188	.307	**.626**	.077
Commenting on a Blog article posted by a non‐British person	.152	.263	**.618**	.171
Looking at an online profile of a non‐British person	−.124	.138	**.558**	.237
Feeling intimidated by a non‐British person passing you on the street	−.047	.133	.228	**.702**
Being in a situation where a non‐British person scares you	.101	.227	.025	**.691**
Making a negative gesture at a non‐British person	.153	.307	.114	**.650**
Actively looking away/ignoring a non‐British person you know well	.035	.042	.076	**.569**
Passing a drunken non‐British person on the street who shouts at you	.031	.286	.313	**.526**
Eigenvalue	7.24	3.66	1.93	1.39
Explained variance	31%	16%	8%	6%
Cronbach’s alpha	.88	.89	.85	.80

#### Traditional contact

This factor accounts for the largest portion of the total variance. It includes eight items, consisting mostly of casual, verbal, face‐to‐face interactions, with familiar outgroup members, such as family, friends, and acquaintances. The majority of interactions described by the items include conversational elements and social activities, such as dining with, or visiting the home of, an outgroup member.

#### Superficial contact

This factor includes five items, consisting mostly of verbal, face‐to‐face interactions in public settings (such as trains, shops, restaurants, or on the street). The items mostly reflect unfamiliar, exposure‐type contact, such as contact with strangers in service interactions.

#### Online contact

The items on this factor relate to online interactions, such as liking, sharing, and commenting on social media posts, as well as looking at someone’s online profile, thus describing contact that is not face‐to‐face and did not take place in a shared location.

#### Negative contact

The items on this factor refer mostly to non‐verbal interactions in adverse situations, resulting from one’s own or an outgroup member’s negative behaviours. They describe situations which involve being scared, intimidated, avoided, or insulted.

### Agreement in the subjective definition of contact

**Figure 1:**
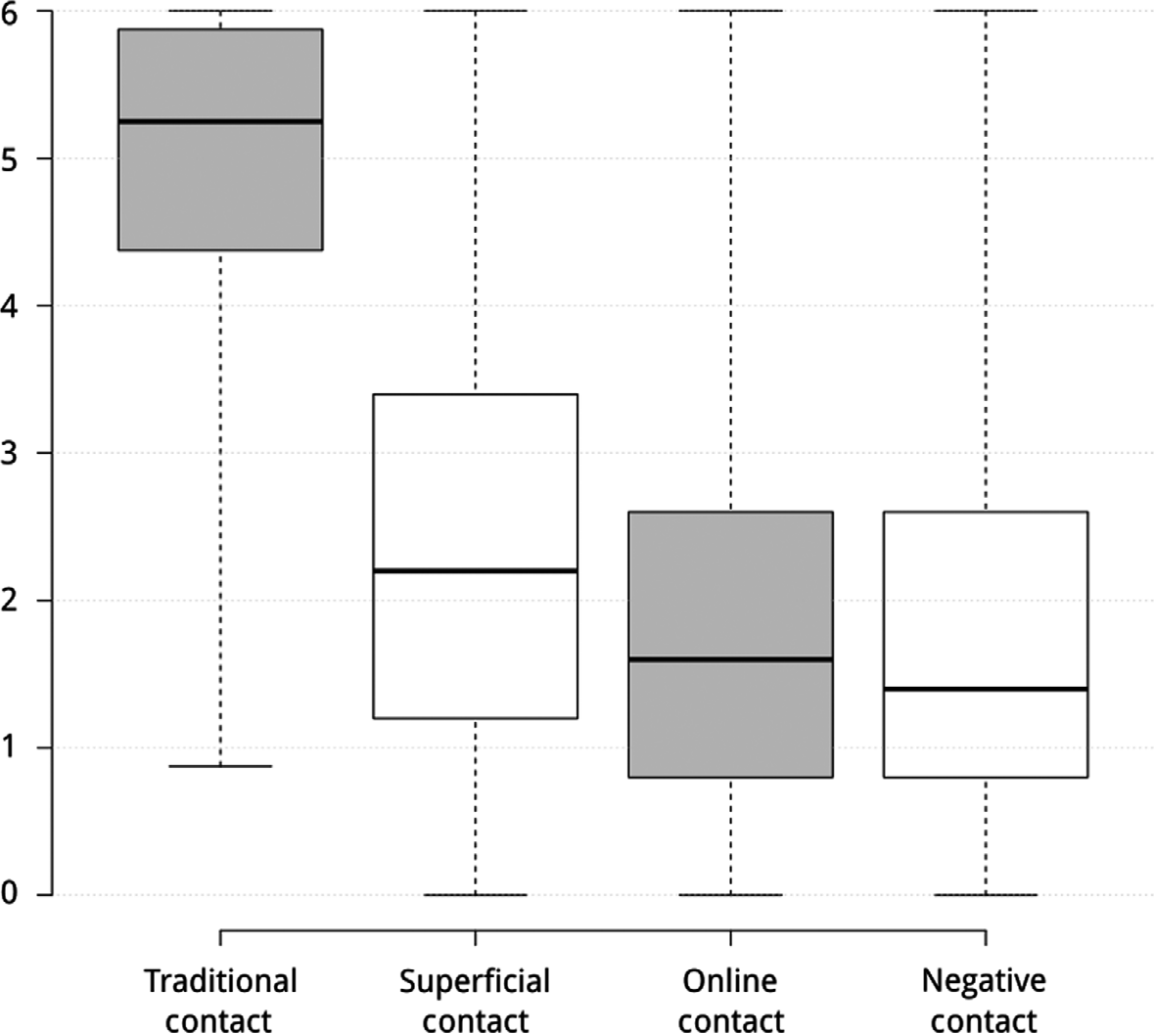
Variations in Subjective Definitions of Different Types of Contact; centre lines show medians; box limits indicate the 25th and 75th percentiles; whiskers extend to minimum and maximum response values; *N* = 498.

Composite scores, based on the mean of the items that primarily loaded onto each factor, were created. Higher scores indicate that contact situations are conceptualized as being more representative of being contact (see Figure 1).

A one‐sample t‐test showed that all factors significantly differed from zero (all *ps* < .001; see Table S2), indicating that all four types of contact were, on average, considered to constitute intergroup contact. Next, a paired‐samples t‐test showed that all factors, except online and negative contact, significantly differed from each other (see Table 3).

**Table 3:** Paired‐samples t‐test between factors

Factors	Δ*M*	Δ*SD*	*t*(497)	*p*	95% CI
Traditional/ Superficial	2.57	1.45	39.45	<.001	[2.44, 2.70]
Traditional/ Online	3.19	1.44	49.49	<.001	[3.06, 3.32]
Traditional/ Negative	3.30	1.47	50.32	<.001	[3.17, 3.43]
Superficial/ Online	0.62	1.27	10.94	<.001	[0.51, 0.73]
Superficial/ Negative	0.73	1.33	12.32	<.001	[0.62, 0.85]
Online/ Negative	0.11	1.37	1.82	.070	[−0.01, 0.23]

Supporting Hypothesis 1, traditional contact was defined significantly more as being contact compared to superficial, online, and negative contact, respectively. Superficial contact was also defined significantly more as being contact compared to online and negative contact. However, there was no significant difference in the definition of online and negative contact as contact. Furthermore, the relatively high mean (*M* = 4.99, *SD* = 0.97, Min = 0.88, Max = 6) and the small interquartile range depicted in Figure 1, suggest a relatively good agreement on traditional contact scenarios as being contact. In contrast, the other three types of contact – contact in superficial settings (*M* = 2.42, *SD* = 1.46, Min = 0, Max = 6), online contact (*M* = 1.80, *SD* = 1.24, Min = 0, Max = 6), and negative contact (*M* = 1.68, *SD* = 1.26, Min = 0, Max = 6) – show a high level of uncertainty and variability regarding their subjective definition as contact. Overall, these findings support Hypothesis 1 that positive, face‐to‐face encounters with known outgroup members are more widely defined as being a contact than superficial, negative, and online contact.

### Correlation analyses

We found a consistent pattern of small but significant positive correlations between participants’ subjective definitions of contact in traditional, superficial, and online contact settings and self‐reported quantity of contact (see Table 4). However, no significant correlations were found between the subjective definition of negative scenarios as contact and self‐reported quantity of contact. Furthermore, only *number of current outgroup friends* did not correlate significantly with the extent to which superficial contact situations are defined as contact. Overall, these findings support Hypothesis 2 that the extent to which situations are subjectively defined as being intergroup contact and the overall amount of self‐reported contact are positively related, with the notable exception of negative contact situations.

**Table 4:** Partial correlations between subjective definition of contact factors and contact quantity

Variables	1	2	3	4	5	6
*Definition of contact*						
1. Traditional	–					
2. Superficial	.34**	–				
3. Online	.17**	.57**	–			
4. Negative	.16**	.53**	.39**	–		
*Intergroup contact quantity*						
5. Amount of past contact	.12**	.10*	.18**	−.03	–	
6. Number of recent contacts^a^	.16**	.11*	.09	−.04	.44**	–
7. Number of current friends^a^	.12**	.01	.10*	−.09	.50**	.44**

a Spearman ρ; controlled for age, gender (male/female), residence (rural/urban), university degree (yes/no); ethnicity (White/BAME)

* *p* < .05 level (2‐tailed)

** *p* < .01 (2‐tailed).

## GENERAL DISCUSSION

Past research has raised a number of issues that intergroup contact research needs to consider, such as attention to the microprocesses of contact (Connolly, 2000), and the consideration of the context specificity of lay constructions of the meaning of contact (Dixon *et al.*, 2005). While some progress has been made, McKeown and Dixon (2017) suggest that there is still a ‘disconnection’ between the kinds of contact on which past research has focused and the participants’ experience of contact in everyday life. The present research addresses one of the issues, namely lay constructions of contact. More specifically, it examines the subjective definition of intergroup contact by asking which situations are considered contact, and the extent to which individuals vary in their definitions.

Findings from Study 1 and 2 show that traditional forms of contact, such as verbal, face‐to‐face interactions with acquaintances, friends, and family are generally defined as being contact. In contrast, encounters with strangers in public, online contact, and negative experiences with outgroup members were defined as contact to a much lesser extent and with more variability in definition.

Overall, participants share the view with intergroup contact researchers that positive, face‐to‐face interactions with known outgroup members constitute intergroup contact (Pettigrew & Tropp, 2006). Traditionally, intergroup contact research has been conducted around such encounters, particularly friendship and acquaintanceship contact. Interestingly, the item ‘Skyping with a non‐British person’ also loaded onto the traditional contact factor rather than the online factor, suggesting that technology‐mediated face‐to‐face contact is defined similarly to physically co‐present face‐to‐face interaction and is distinct from other computer‐mediated forms. As indicated by findings from Study 1, some social media platforms and communication technologies appear to be reserved for interactions with well‐known others and are seen as distinct from online platforms that focus on interactions with strangers.

In both Study 1 and Study 2, participants did not share an agreed definition of contact when it comes to superficial interactions with strangers as well as online interactions. Answers in Study 2 ranged from ‘not at all’ to ‘very much’, reflecting the disparate nature of lay constructions of contact at the lower boundary found in Study 1. This finding suggests that the assessment of superficial contact is not only difficult due to memory effects (Barclay, 1986; Pachur, Schooler, & Stevens, 2013; Schwarz & Sudman, 1994) but also because participants do not universally define superficial encounters as ‘contact’. This finding may help to shed light on differences in empirical results between intergroup contact research and urban research on the effects of diversity on intergroup relations (see Wessel, 2009, for an overview). Whereas the contact literature shows that more opportunities for contact lead to more contact which, in turn, reduces prejudice (e.g., Wagner *et al.*, 2006), the urban literature suggests that ethnic mixing in neighbourhoods is characterized by a drive for self‐segregation (see Durrheim & Dixon, 2006). These research traditions, however, tend to use different methods and have different conceptualizations of ethnic mixing. In contrast to self‐report measures of (mostly) face‐to‐face encounters with relatively well‐known others, urban research uses neighbourhood statistics and observational research, conceptualizing mixing as physical co‐presence. As our findings indicate, relatively superficial encounters are less likely to be considered by participants as ‘contact’ and are therefore less likely to be self‐reported than traditional face‐to‐face contact. Where little incentive for interactions exist, physical co‐presence is likely to be characterized by superficial encounters, reducing the comparability of results across traditions. 

A finding that had not been foreshadowed by the interview findings in Study 1 is the lack of agreement on whether negative situations constitute intergroup contact. In Study 1, a few participants provided examples of negative contact when asked about recent intergroup contact. In contrast, Study 2 results suggest that contact is more typically defined by lay people as a positive rather than a negative interaction. This result is surprising as past research suggests that negative encounters are more memorable and can be more influential than positive contact (Barlow *et al.*, 2012; Graf *et al.*, 2014).

Our research also points to the importance of ‘locatedness’ in the subjective definition of contact by showing that participants take the location in which a contact occurs into account when assessing the encounter (Dixon, 2001; Dixon *et al.*, 2020; Durrheim & Dixon, 2006; Tredoux, Dixon, Underwood, Nunez, & Finchilescu, 2005).

Together, these findings show that a shared definition of contact cannot be assumed by researchers, particularly when studying contact with strangers, online contact, or negative contact. Similarly, disparate assumptions on the boundaries of contact need to be taken into account when discussing contact interventions with stakeholders and when designing interventions that go beyond workshops with relatively small groups, such as urban design that enhances contact opportunities, and online interventions.

Disparate subjective definitions of contact also threaten the validity of standard self‐report measures that are widely used in the contact literature: Participants who define more situations as being contact report more contact without necessarily having experienced more actual contact. Our findings provide initial evidence of such conceptual inflation: They reveal a statistically significant, positive relationship between the extent to which different scenarios were defined as being contact and the amount of contact participants self‐reported. Although correlations were small, they were consistently positive across traditional, superficial, and online settings. Here, it needs to be noted that the small‐to‐moderate size of the correlation between subjective definitions of contact and self‐reported contact is comparable to the mean correlation of ‐.22 between self‐reported contact and prejudice reported in the meta‐analysis by Pettigrew and Tropp (2006). Furthermore, our relatively large sample is likely to be more heterogeneous than most samples in the contact literature (e.g., schoolchildren, exchange programme/language school students, university students, participants in a contact intervention of a well‐defined ethnic group). Correlations tend to be stronger in more homogeneous samples.

### Limitations and future research

Our findings provide initial evidence that a shared definition of contact – particularly at the lower boundary – cannot be assumed by intergroup contact researchers. Future research needs to establish (1) whether conceptual inflation is a threat to self‐report contact measures other than the common ones reported here, as well as to quality of contact measures, and (2) the effect this has on the contact–prejudice relationship.

Given the cross‐sectional nature of Study 2, it is unclear whether the problem is one of conceptual inflation, or whether the causal effect is reversed: Do those who experience more intergroup contact subsequently consider a wider range of experiences as being contact? In this case, the experience of intergroup contact would be widening the horizon for alternative definitions of intergroup contact, in line with the deprovincialization argument by Pettigrew (1997). Longitudinal or experimental research is needed to test whether the same amount of actual contact is defined and then reported, as quantitatively (and qualitatively) different, in line with different subjective definitions of contact, or whether more experienced contact leads to a widening of the subjective contact definition, or both.

Future research may also wish to explore the reasons for differences in subjective definitions of contact, and the role that cultural norms play. Some of the variation in the subjective definition of intergroup contact may stem from different cultural norms that participants adhere to. For instance, eye contact during social interactions is experienced differently in Eastern and Western cultures, particularly between strangers. While Western cultures encourage eye contact during interactions, Eastern cultures tend to experience the maintenance of eye contact as disrespectful (Argyle, Henderson, Bond, Iizuka, & Contarello, 1986). Furthermore, such cultural differences have been found to affect person evaluations, with Japanese participants rating faces as angrier, and less pleasant, than Finnish participants when making eye contact (e.g., Akechi *et al.*, 2013). However, it needs to be noted that we largely replicated findings from Study 1 with an ethnically homogeneous British sample in Study 2, thereby limiting an explanation based on cultural factors alone. Furthermore, the correspondence of findings between Study 1–where we used a culturally diverse sample and qualitative methods–and Study 2–where a survey study was employed with a culturally homogeneous majority group sample–suggests that our findings are robust across cultural groups and methods, for minority and majority groups.

In addition, future studies may wish to consider a wider range of contact scenarios to assess subjective definitions of contact. For instance, in Study 1, participants were prompted by the researcher to consider particular encounters (e.g., online contact) or certain aspects of a situation (e.g., eye contact) if they had not mentioned these already. These prompts were based on definitions of social interactions. However, there are many other aspects and contexts that might be interesting to explore in future research, such as making space for someone else (e.g., in a crowd, on public transport).

In Study 2, we included several encounters from Study 1 but also a range of other possible contact scenarios. However, the breadth of scenarios meant that each scenario was described in very minimalistic terms. As participants in Study 1 pointed out, it is often a particular context or relationship as well as the quality of the interaction (e.g., a meaningful conversation or other aspect) that can turn a mundane activity (e.g., shopping, boarding a bus) into a recognized intergroup contact encounter. As research on imagined contact has shown, more elaborate scenarios may yield stronger results than short scenarios (Husnu & Crisp, 2010). Future research is therefore needed to understand the extent of agreement on a particular contact encounter by using fewer but more detailed descriptions of contact scenarios. Given the results on negative contact and its relevance for research on the contact–prejudice link, it would be particularly fruitful to investigate which kind of negative situations are considered contact and the reasons for their inclusion or exclusion.

Another important area for future research is the extent to which subjective definitions of contact affect the relationship between self‐reported intergroup contact and intergroup outcomes, particularly prejudice. While our research points towards an effect of definition on self‐reported contact, we did not examine the contact–prejudice relationship. Furthermore, in addition to contact quantity, self‐reported quality may also be affected if some participants define contact as including fleeting and negative encounters, whereas others only report positive and meaningful experiences. Future work should therefore also include a measure of contact quality.

### Conclusion

The studies reported here provide first evidence that the subjective definition of situations as intergroup contact is complex and multifaceted. Most importantly, results from Study 1 illustrate that subjective definitions of what characterizes contact, especially in regard to more superficial types of contact, vary greatly. Study 2 replicates these findings in a quantitative study showing a positive relationship to self‐reported intergroup contact. This suggests that a universally shared definition of contact cannot be assumed when measuring contact beyond positive, face‐to‐face encounters with friends and acquaintances.

Given the changing focus of intergroup contact research away from face‐to‐face friendship contact, researchers need to consider whether their own definition of contact can be assumed to be shared by participants or other stakeholders with whom they communicate. With regard to measurement, overly vague items may not serve the specific research purpose well and may need to be supplemented with more clearly specified items. The danger here, of course, is that an overly prescriptive measure misses relevant contact experiences. However, we believe that the findings presented here can help researchers to better reflect on subjective definitions of contact, and the extent to which they may affect their research and impact work.

## Conflicts of interest

All authors declare no conflict of interest.

## Supporting information


**Appendix S1.** Material.

## Data Availability

The data that support the findings of this study are openly available in OSF at https://doi.org/10.17605/osf.io/u5qj7, reference number U5QJ7.
