# Supplementary material for: Variations in subjective definitions of everyday situations as intergroup contact
Source: Br J Soc Psychol. 2020 Feb 24;59(4):965–91. doi: 10.1111/bjso.12372 (PMC7586920; doi:10.1111/bjso.12372)
Supplement: Supplementary file 1 — Appendix S1. Material. [file BJSO-59-965-s001.docx]

**Supplementary Material**

**Instructions given to Participants for Study 1**

**Procedures**

This study consists of two parts, a contact diary and an interview, as outlined below:

**1. Contact diary:** This first part of the study will require you to keep a record of any contact that you have with anyone of a different nationality to your own. This task should be carried out over a period of three consecutive days; one of the three days must be a weekend/bank holiday day. You may choose one time period below (please choose one that suits you best):

1) Thursday, 22nd May - Saturday, 24th May until 23:59

or

2) Monday, 26th May - Wednesday, 28th May until 23:59

Please keep a record of every contact you have during this time. You can record this information any way you like. If possible, please also note down the date and time of each contact.

**2. Interview:** After the first part of the study has been completed, you will be required to take part in an interview. The interview will be an open-ended, informal conversation about your experiences during the contact diary phase of the study. It will last at least 30 minutes but not longer than one hour. Please bring along the notes you made during part one. The interview will be audio-recorded and during the interview the researcher may take notes.

Table S1
*Perceived Contact Scenarios (67 items, shown in random order; Study 2)*

| **Contact Scenario Item** | **Source** |
| --- | --- |
| Being in a situation where a non-British person scares you | p. 393 / 572 |
| Interacting with a non-British homeless person on the street | p. 219 / 518 |
| Passing a drunken non-British person on the street who shouts at you | p. 437 / 835 |
| Smiling at a non-British person | p. 378 / 211 |
| Chatting informally to a non-British work colleague | p. 298 / 212 |
| Making a service call (phone) to a non-British person | p. 97 / 828 |
| Attending a social gathering mainly frequented by non-British people | p. 160 / 536 |
| Interacting with a non-British person seeking support for a political campaign |  |
| Being in a situation where a non-British person is angry with you | p. 346 / 837 |
| Making eye-contact with a non-British person | p. 229 / 129 |
| Chatting on the phone to a non-British family member | p. 397 / 639 |
| Requesting help from a non-British person (e.g., asking for directions) | p. 160 / 520 |
| Looking at/observing a non-British person for a longer time | p. 219 / 512 |
| Daily interaction with a non-British friend or acquaintance | p. 59 / 439 |
| Playing an online game with a non-British person | p. 21 / 522 |
| Chatting on the phone to a non-British friend or acquaintance | p. 45 / 73 |
| Nodding your head at a non-British person, as a greeting | p. 351 / 93 |
| Giving a presentation to a non-British group of people |  |
| Participating in a forum discussion with non-British people |  |
| Having an informal conversation with a non-British person you know | p. 106 / 215 |
| Interacting with a non-British person in a meeting/class |  |
| Daily interaction with a non-British colleague at work | p. 296 / 164 |
| Polite behaviour, e.g. holding a door open for a non-British person |  |
| Interacting with a non-British customer/client at work | p. 201 / 65 |
| Sitting in a group of non-British people without interacting | p. 6 / 130 |
| Informal interaction with a subordinate non-British person at work |  |
| Sending a non-British person a Birthday or Christmas card | p. 337 / 588 |
| Texting a non-British person | p. 446 / 1047 |
| Taking a photo of a non-British person/group whom you don't know | p. 443 / 719 |
| Skyping with a non-British person | p. 44 / 71 |
| Having a drink with a non-British person you meet by chance at the pub | p. 525 / 512 |
| Commenting on a Blog article posted by a non-British person | p. 447 / 1097 |
| Making a negative gesture at a non-British person |  |
| Looking at an online profile of a non-British person |  |
| Purchasing a ticket from a non-British person on a train or bus | p. 200 / 46 |
| Going out to dinner with a non-British person | p. 319 / 102 |
| Being interviewed for a job by a non-British person | p. 240 / 423 |
| Intervening in an argument between non-British strangers on the street | p. 420 / 376 |
| Shouting at a non-British driver who has obstructed you in some way |  |
| Thinking intensely about a non-British person who is not present |  |
| Having a brief chat to a non-British person you meet by chance | p. 307 / 404 |
| Haggling with a non-British shop owner | p. 14 / 362 |
| Giving money to a non-British street artist performing in public |  |
| Commenting on a Facebook/Twitter post from a non-British person | p. 129 / 261 |
| Receiving a marketing phone call from a non-British person |  |
| “Liking” a post on Facebook/Twitter from a non-British person | p. 104 / 170 |
| Actively looking away or ignoring a non-British person you know well | p. 293 / 593 |
| Interacting with a non-British person at a night club | p. 274 / 92 |
| Talking to your non-British neighbour | p. 83 / 491 |
| Being in a situation where a non-British person smiles at you | p. 293 / 96 |
| Interacting with a non-British person seeking support for a charity | p. 151 / 278 |
| Chatting to non-British table neighbours in a restaurant |  |
| Ordering something to drink or eat from a non-British person | p. 150 / 267 |
| Visiting your non-British friend’s or acquaintance's home | p. 68 / 122 |
| Having lunch at a cafeteria with a non-British person |  |
| Arranging an appointment with a non-British person |  |
| Giving way to a non-British person who is passing you |  |
| Giving money to a non-British person who is begging | p. 220 / 546 |
| Being served by a non-British person in a shop or supermarket | p. 114 / 422 |
| Meeting with a non-British superior at work/university | p. 103 / 139 |
| Helping a non-British person on the street to light their cigarette |  |
| Giving a non-British person whom you don't know a lift |  |
| Talking to a non-British person in an online chat | p. 20 / 499 |
| Sharing/retweeting a non-British person's post on Facebook/Twitter | p. 104 / 168 |
| Briefly greeting a non-British person (verbally) | p. 29 / 154 |
| Writing an email to a non-British person | p. 254 / 240 |
| Feeling intimidated by a non-British person passing you on the street | p. 353 / 156 |

*Note:* Items without a source where generated by the

authors. Page and line numbers refer to the interview

transcript document of Study 1, available at <https://doi.org/10.17605/osf.io/u5qj7>

Table S2
*One-sample t-test of perceived contact factors against zero (Study 2)*

| Factors | *M* | *SD* | *t(497)* | *p* | 95% CI |
| --- | --- | --- | --- | --- | --- |
| Traditional contact | 4.99 | 0.97 | 115.06 | .000 | [4.90, 5.07] |
| Superficial contact | 2.42 | 1.46 | 36.96 | .000 | [2.29, 2.54] |
| Online contact | 1.80 | 1.24 | 32.30 | .000 | [1.69, 1.90] |
| Negative contact | 1.68 | 1.26 | 29.89 | .000 | [1.57, 1.79] |
